# Is the hibiscus harlequin bug aposematic? The importance of testing multiple predators

**DOI:** 10.1002/ece3.914

**Published:** 2013-12-15

**Authors:** Scott A Fabricant, Carolynn L Smith

**Affiliations:** 1 Department of Biological Sciences, Macquarie UniversityNorth Ryde, NSW, 2109, Australia; 2 Department of Environment and Geography, Macquarie UniversityNorth Ryde, NSW, 2109, Australia

**Keywords:** Aldehydes, aposematism, avoidance learning, chemical defenses, heteroptera, *Tectocoris*

## Abstract

Aposematism involves predators learning conspicuous signals of defended prey. However, prey species utilize a wide range of chemical (or physical) defenses, which are not likely to be equally aversive to all predators. Aposematism may therefore only be effective against a physiologically sensitive subset of potential predators, and this can only be identified through behavioral testing. We studied the emerging model organism *Tectocoris diophthalmus* (Heteroptera: Scutelleridae), an aposematically colored but weakly defended shieldback stinkbug, to test the efficacy of its defenses against a suite of predator types. We predicted the bugs' defenses would be ineffectual against both experienced and naïve birds but aversive to predaceous insects. Surprisingly, the opposite pattern was found. Both habituated wild passerines and naïve chickens avoided the bugs, the chickens after only one or two encounters. To avian predators, *T. diophthalmus* is aposematic. However, praying mantids showed no repellency, aversion, or toxicity associated with adult or juvenile bugs after multiple trials. Comparison with prior studies on mantids using bugs with chemically similar but more concentrated defenses underscores the importance of dose in addition to chemical identity in the efficacy of chemical defenses. Our results also emphasize the importance of behavioral testing with multiple ecologically relevant predators to understand selective pressures shaping aposematic signals and chemical defenses.

## Introduction

Aposematism is the phenomenon in which defended prey advertise their unprofitability through conspicuous signals, such as bright coloration, pungent smells, or harsh sounds (Rowe and Guilford 1999). Conspicuousness to predators has been shown to enhance identification, learning, and memory in the predators (reviewed in Ruxton et al. 2004). Many species that exhibit conspicuous traits (often bright or contrasting colors) in conjunction with potentially noxious chemicals are labeled aposematic. However, this label is often applied without conducting behavioral tests (Bernays et al. 1977; Moore and Brown 1981; Staddon et al. 1987; Williams et al. 2001; Schwarz et al. 2009). Previous research has shown that not all species that exhibit aposematic coloration use this to defend against predators (Talianchich et al. 2003). Other common uses include sexual displays (Metz and Weatherhead 1991), nonsexual intraspecific communication (Papaj and Newsom 2005), mimicry (Brodie and Howard 1973), and startle or disruptive coloration (Stevens 2005; Stevens et al. 2006). It is hence important to explore the possible functions of conspicuous coloration in an organism.

Aposematic signaling is only beneficial if the defenses are effective against would-be predators. The efficacy of chemical defenses may also be different for specific groups of predators (McIver and Lattin 1990; Exnerová et al. 2007). For birds, defenses that are emetic-or illness-inducing should lead to stronger and more persistent avoidance learning compared with distasteful or irritating defenses (Alcock 1970). Literature suggests that many heteropteran chemical defenses, particularly short-chain aldehydes produced from exocrine glands, should act as nonspecific irritants with low effectiveness against birds (Staddon 1979; Aldrich 1988). However, there is also evidence to suggest that endogenously produced irritants in some heteropteran species have varying degrees of success in repelling birds, even on first attack (Schlee 1986; Staples et al. 2002; Svádová et al. 2010; but see Alcock 1973). Aldehydes have been shown to be effective deterrents to arthropod predators, including ants (Remold 1963) and mantids (Noge et al. 2012). Exposure to aldehydes, particularly keto-aldehydes, in higher concentrations can result in paralysis or death (Prudic et al. 2008; Eliyahu et al. 2012). This differential efficacy underscores the importance of specifically testing each presumably aposematic species with ecologically relevant predators using behavioral experiments.

**Figure 1:**
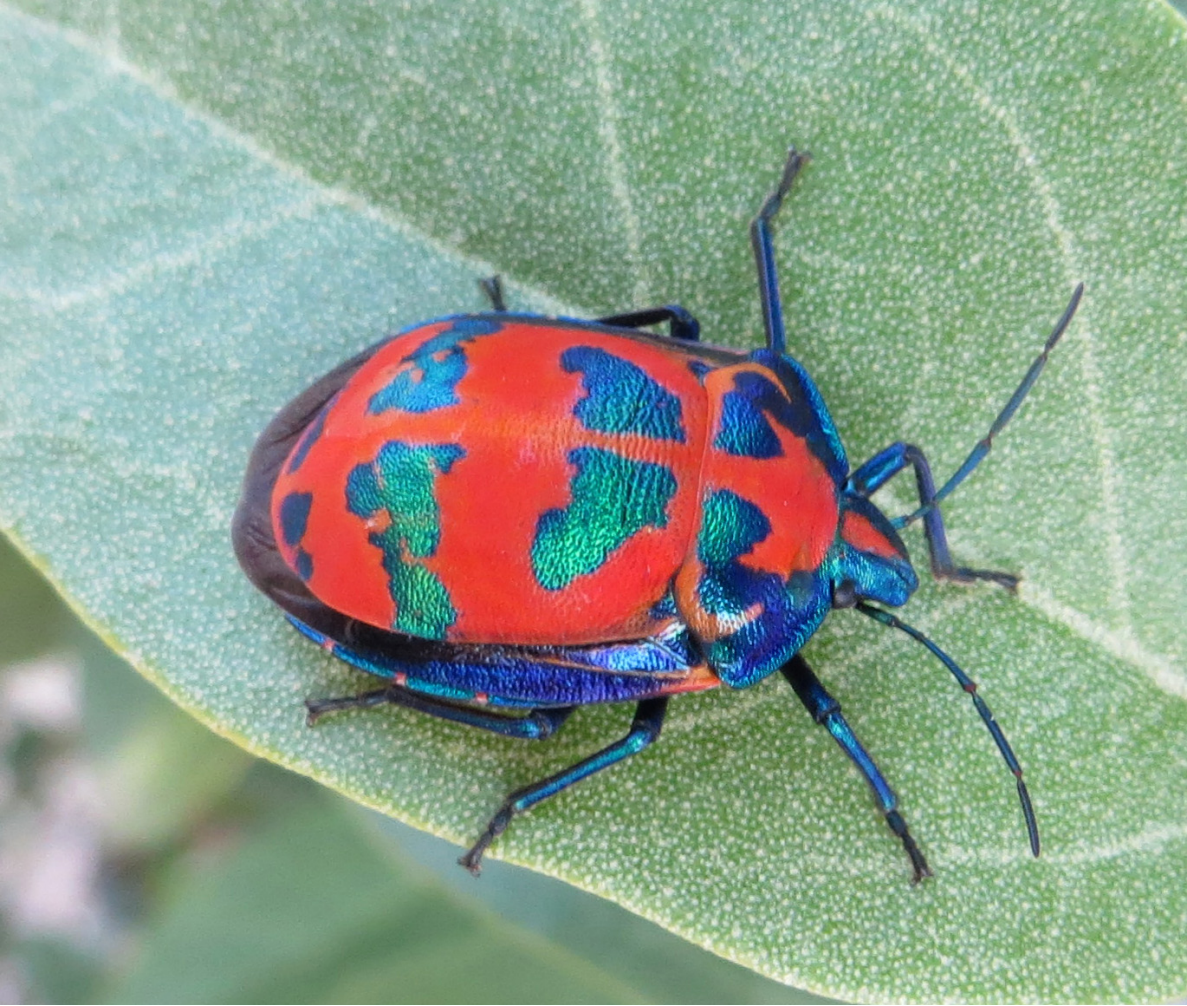
Image of a typical hibiscus harlequin bug *Tectocoris diophthalmus* (Scutelleridae) foraging on a *Lagunaria patersonia* (Malvaceae) tree in Narrabeen, NSW.

The hibiscus harlequin bug, *Tectocoris diophthalmus* (Heteroptera: Scutelleridae), is a large, brightly colored member of the Australian jewel bug fauna and has emerged as a useful study system for questions of behavioral ecology (Ballard and Holdaway 1926; Wilson et al. 1983; Hoese et al. 2006; Fabricant et al. 2013) and chemical signaling (Schaefer 1972; Smith 1978; Knight et al. 1985; Staddon et al. 1987). The bug features iridescent blue patches on a bright orange background, and adults have an enlarged scutellum covering their entire dorsal surface (Fig. 1). Females guard eggs in exposed positions for up to 3 weeks, which suggests a possible function for defensive chemicals and aposematic coloration (Ballard and Holdaway 1926). Its glandular secretions have been identified as being primarily short-chain aldehydes and alkanes, with additional production of keto-aldehydes in juveniles. These secretions are released from metathoracic glands in adults or dorsal glands in juveniles, and there is no histological or chemical evidence of accessory glands or other internal storage for sequestered plant toxins (Staddon et al. 1987). While a number of studies have explicitly labeled *T. diophthalmus* as aposematic based on the presence of bright coloration and putatively noxious secretions, these same studies have also demonstrated that the total output of defensive secretions is very small and dispersal structures are poorly developed (Schaefer 1972; Smith 1978; Staddon et al. 1987), suggesting that the bug may be poorly defended against predators. To date, no study has actually tested the defenses of *T. diophthalmus* against predators.

Our aim was to test the efficacy of the defenses of *T. diophthalmus* in inducing aversion and/or avoidance learning in avian and arthropod predators. Based on previous research, we hypothesize that *T. diophthalmus*, which utilizes aldehyde-based defenses, should be either weakly defended or undefended against birds (Alcock 1973; Aldrich 1988) and unlikely to induce avoidance learning. Conversely, the bugs should be strongly defended against invertebrates (Remold 1963; Eliyahu et al. 2012), potentially inducing avoidance learning. We assayed aposematic efficiency in both wild populations and controlled experiments. The use of wild populations permits an estimate of survival in typical habitat and conditions. Conversely, the use of naïve predators allows us to eliminate the influence of generalization, wherein prior experience with conspicuous unpalatable prey results in unlearned avoidance by similarity (Hotová Svádová et al. 2013). In controlled conditions, we can identify whether any aposematic effects are due to innate wariness, unlearned biases, or learned avoidance.

## Methods

### Ethical note

All bird experiments were carried out with the permission of the Macquarie University Animal Ethics Committee, Animal Research Authority (ARA) Number 2011/060. Unless otherwise stated, bugs used in experiments were adult, *Tectocoris diophthalmus*, harlequin bugs collected from Narrabeen, NSW, a suburban area next to the beach, northeast of Sydney, NSW. No special permissions are required for the collection of this species or from this site.

### Experiment 1: efficacy of defense against wild birds

We tested the efficacy of *T. diophthalmus* defenses on wild birds using feeding trays baited with locally caught bugs. Using locally caught bugs, we increased the likelihood that birds participating in the test had prior experience with them. This portion of the study was conducted at two sites in Narrabeen, New South Wales, Australia, approximately 3 km apart (Site 1: 33.722587 S, 151.295533 E; Site 2: 33.749532 S, 151.291692 E), starting on 19 March 2012. The test apparatus consisted of four 43-cm-diameter mesh-bottom hanging feeder trays that were lined with leaves from Norfolk Island Hibiscus (*Lagunaria patersonia*) using Blu-Tack (Bostik, Paris, France). The trays were paired to create replicates at each of the two sites. Each pair was secured to low hanging branches of a *L. patersonia* tree by 50 cm chains approximately 3 m apart within the same tree. To acclimate wild birds in the area to the feeders, each feeder had 10 mealworms (*Tenebrio* sp. larva) attached to the leaves using a small drop of cyanoacrylate glue. Missing or dead mealworms were replenished with new mealworms at 24 and 48 h after placement.

On day 4, 40 adult *T. diophthalmus* were collected from trees of *L. patersonia* near Site 1. Ten were secured to each tray using a small drop of cyanoacrylate glue on the abdomen, avoiding the metathoracic glands. Each site was observed for 1 h for evidence of bird visitation (at 1200 and 1330). The trays were then surveyed 24 h later for live, dead, and missing bugs. Trays were then removed from the sites.

### Experiment 2: efficacy of defense against naïve chickens

We used eight male golden Sebright bantam adult chickens (*Gallus gallus domesticus*) to test the response of naïve birds to the bugs' defenses. The birds were housed at Macquarie University as part of a breeding stock for other experiments under ARA 2009/057. Each bird was individually marked with a unique colored and numbered band. During the experiment, birds were kept in pairs to reduce stress of captivity and experimentation on this social species. After cessation of the experiments, the chickens were returned to their previous flocks. Subjects had no prior experience with *T. diophthalmus*.

On 10 December 2012, pairs of birds were placed in test enclosures (5.3 × 1.3 × 2.4 m, l × w × h), constructed of chain link fencing. The ground substrate was dirt and mulch, and enclosures included a covered refuge with perches and straw for nesting. Chickens were given *ad libitum* commercial pellet food (Gordon's Commercial Laying Ration, Sydney, Australia) and water. The chain link fence between enclosures allowed visual and auditory communication, although shade cloth disrupted visual contact during testing procedures.

Chickens were given 4 days to habituate to the test aviaries, followed by 4 days of acclimation trials. All trials were conducted between 1500 and 1700. During the acclimation trials, four mealworms were glued with a small drop of cyanoacrylate glue to a wood block (40 × 11 × 2 cm, l × w × h), equidistant along the long axis. The cage door was opened, and then, the camera (Canon Powershot SX260 digital; Canon Australia, North Ryde, NSW, Australia) was placed on a tripod at ground level and recording was started. We then placed the tray on the ground, closed the enclosure door, and stepped away from the enclosure. Trials ended when both chickens had move away from the tray and began engaging in other behaviors not directed toward the tray.

Chicken groups have stable dominance hierarchies and individual behavior varies by rank (Davis and Domm 1943). In these groups, alpha males gain priority access to ephemeral food sources when discovered (McBride et al. 1969). During the current test conditions, this created a condition wherein the beta male had the opportunity to observe the alpha male interacting with the bugs prior to having access to them. Previous research reveals that viewing a companion feeding from or rejecting a feeding location alters the preference of the observer (Nicol 2006). We hence expected that the alpha males and beta males might have different responses to the bugs. In addition to acclimating to the testing procedure, these acclimation trials allowed us to determine the alpha male in each pair, based on which male ingested the majority of the mealworms from the trays.

The test trials were identical to the acclimation trials with the exception that four *T. diophthalmus* bugs, rather than mealworms, were secured by gluing three of six legs with a drop of cyanoacrylate glue to the blocks. The color pattern and sex of the bugs on each tray was randomized to represent the natural variation in bugs to which a free-living predator would be exposed. Trays were presented to the birds in rounds, with each pair of birds receiving one tray per round. Each pair completed the trial before the next pair was tested. Each round had a total of 16 bugs available (four bugs per tray by four pairs of birds). On the first day of testing, each pair of chickens was given a tray of four bugs (round 1). After a 15 min interval, a second tray of four bugs was presented (round 2). This was designed to test one-trial learning. To test longer-term memory, each pair of chickens was given another round of four bugs at 72 h (round 3) and another 72 h later, equivalent to 144 h after round 1.

Videos were scored at half speed (40 frames per s; PAL standard) using VLC (version 2.0.5; VideoLAN, Paris, France). Attacks were scored if a chicken contacted an individual bug with its beak. Only the first contact by any chicken on any bug was scored. Data were analyzed using number of bugs attacked per chicken per round using a linear mixed effects model, with bird identity nested within pair as a random effect, and dominance rank (alpha or beta) and round (1–4) as fixed effects. Residuals were tested for normality and checked for pattern against fitted values. For significant interaction terms, simple main effects were calculated using conditional means and full-model error. Adjustment of *P*-values for *post hoc* pairwise comparisons was conducted using Benjamini and Hochberg's (1995) false discovery rate method, which balances the likelihood of type I versus type II errors when conducting multiple pairwise corrections (Benjamini and Hochberg 1995; Verhoeven et al. 2005). Statistics were performed with SPSS for Windows v.20 (IBM, Armonk, NY).

### Experiment 3: efficacy of defense against mantids

We tested the efficacy of *T. diophthalmus* defenses against arthropods using *Hierodula majuscula* (Mantidae) mantids. Female *H. majuscula* (*n* = 13) were purchased from Minibeast Wildlife (Kuranda, QLD, Australia) and reared to adulthood on a diet of crickets (*Acheta domestica*) supplied by Pisces Enterprises (Brookfield, QLD, Australia). They were kept in separate enclosures (24 × 20 × 15 cm, l × w × h), with sides and top partially constructed from plastic mesh, and kept in a 27°C room with 14 h:10 h photoperiod. Before trials began, mantids were weighed with a digital scale and had their pronotum measured with digital calipers. The residuals of weight regressed against pronotum length were calculated as a proxy for condition (Jakob et al. 1996). Food was withheld from mantids for 72 h to help ensure feeding motivation. Trials began 11 March 2013 and were conducted between 1400 and 1800.

In the trials, a mantid was placed on a green piece of paper, facing 90° away (side randomized) from the starting line for the bug. One bug was released from the starting line, 20 cm from the mantid, facing it. If the bug crawled away from the mantid, the bug was returned to the starting line and the timer restarted. A stopwatch was started when the mantid swiveled its head to face the bug. This behavior was used as an indication of attention to the bug. Bugs showed no behavioral indicator of being oriented toward the experimenter's hand when being placed in the arena. Time was measured between attention and strike with raptorial forelimbs, and from strike to end of consumption. The sex of the bug used in any given trial was randomized. Bugs were weighed before the trials, and any pieces of bug remaining after feeding were weighed as well. Each mantid was tested with one bug per day, repeated every 24 h for 4 days. Based on previous research (Berenbaum and Miliczky 1984; Bowdish and Bultman 1993; Prudic et al. 2007), 4 days was chosen as sufficient period for mantids to learn to recognize aposematic signals. Data were analyzed using a Friedman's test, with time to strike or weight remaining as response variables and mantid identity as the blocking factor. Statistics were performed on SPSS for Windows v.20.

To test for the possible enhanced avoidance learning of keto-aldehydes, tests were also performed using last-instar juvenile *T. diophthalmus*, which produce a keto-aldehyde and alkane in addition to aldehydes (Staddon et al. 1987). This portion of the study was performed using four wild-caught female *Pseudomantis albofimbriata* (Mantidae). This species is smaller than *H. majuscule*, which may increase the likelihood of negative reactions to the noxious secretions. The mantids were collected from a suburban area in West Pymble, New South Wales (33.758434 S, 151.134150 E). Due to differences in vegetation, it is highly unlikely that these mantids had had experience with *T. diophthalmus*, and so were likely naïve predators. Trials with *P. albofimbriata* began 7 May 2012. Each *P. albofimbriata* mantid was offered one last-instar juvenile *T. diophthalmus* per day for 4 days using forceps. Response was scored as ignore, strike and reject, partial consumption, or total consumption (pieces of legs dropped were still considered total consumption). Due to low sample size, no statistical analysis was performed.

## Results

### Experiment 1: efficacy of defense against wild birds

By the third day, all 10 mealworms were removed from all trays within the 24-h period, which indicated that the birds had acclimated to the trays. In the hour-long observation periods after restocking with harlequin bugs, two birds (Noisy Miners, *Manorina melanocephala*) visited the pair of trays at Site 1 on independent occasions, and one bird of the same species visited the pair of trays at Site 2. In all three instances, birds landed on and examined both trays before flying off. No bugs were taken or damaged in these visits.

No bugs were removed from any of the trays or showed signs of bird-induced damage after 24 h. Most individuals were still alive, although a few were either dead or nonresponsive to touch. Two bugs near the middle of one tray at Site 1 had been severely damaged (i.e., complete removal of internal body parts) by ants. It could not be determined whether the ants killed these bugs or scavenged upon them. All bugs that were alive on the tray at the time of assessment were undamaged by the ants.

### Experiment 2: efficacy of defense against naïve chickens

During the acclimation trials, pairs exhibited a stable dominance hierarchy in which the same male consistently approached and interacted with the feeding trays first. This individual consumed the majority of mealworms from each training tray. The second male had limited access to the trays until after the first male ceased interacting with the trays. This pattern continued throughout the training and test trials. Based on this stable behavior, we designated one male as the alpha male and the other as the beta male.

**Figure 2:**
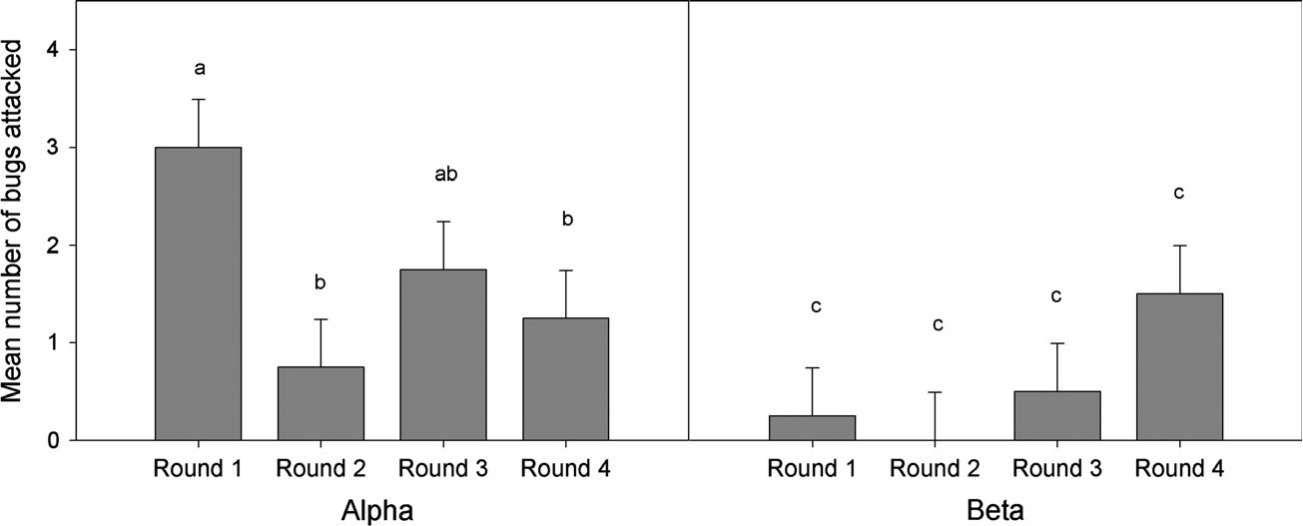
Mean number of bugs attacked per round by chickens. The first two rounds occurred 15 min apart on day 1, while rounds 3 and 4 occurred 72 and 114 h (3 days intervals) after round 1. The four bars on the left are for the dominant “alpha” males (*n* = 4), while the bars on the right are for submissive “beta” males (*n* = 4). Error bars are standard error of the mean. Letters indicate significant differences (*P* < 0.05) after false discovery rate correction.

Overall, there was a significant interaction between rank and round (*F*_3,18_ = 4.839, *P* = 0.01), in addition to significant main effect for round (*F*_3,18_ = 3.613, *P* = 0.003) and marginally significant effect for rank (*F*_1,6_ = 5.341, *P* = 0.06). To account for the significant interaction, we compared the two ranks separately with simple main effects tests. We found a significant effect of round for the alpha males (*F*_3,18_ = 5.774, *P* = 0.006), but not for the beta males (*F*_3,18_ = 2.677, *P* = 0.8). In pairwise comparisons corrected using the false discovery rate, there were significant reductions in attack rate for alphas between round 1 and 2 (FDR-adjusted *P* = 0.012), and between round 1 and 4 (FDR-adjusted *P* = 0.036). No other pairwise comparisons were significant (Fig. 2).

Of a total of 64 bugs offered to chickens, and a total of 36 bugs attacked, only two bugs were consumed by a chicken. These two bugs were consumed by the same beta chicken, in the same round (round 4). Only one other bug was damaged to the point of opening the body cavity. The rest were either left in place on the trays or picked up and dropped.

### Experiment 3: efficacy of defense against mantids

**Figure 3:**
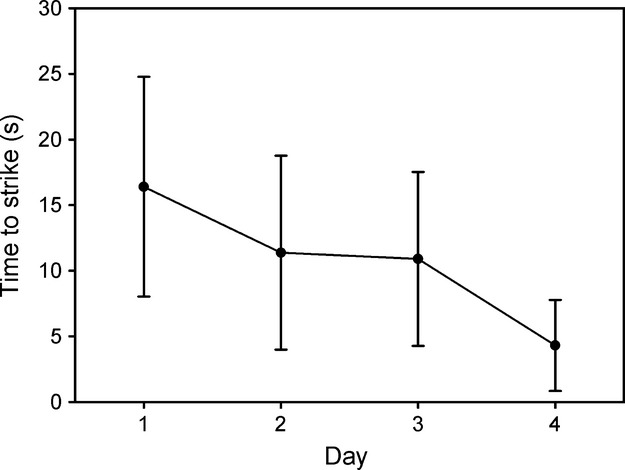
Latency for *Hierodula majuscula* mantids to attack harlequin bugs. Mean length of time (s) ±95% CI from first orientation of the mantid (*n* = 13) toward the bug until capture of the bug, over the 4 days of feeding trials.

All *Hierodula majuscula* mantids struck every time they were presented with an adult harlequin bug on all 4 days. The latency time between attention and striking decreased significantly with experience (Friedman's Test: *Q* = 13.214, df = 3, *P* = 0.004, two-tailed; Fig. 3). The total weight of bug material consumed did not change over the course of the trials (Friedman's Test *Q* = 2.077, df = 3, *P* = 0.56, two-tailed).

In the experiment with juvenile bugs, all *Pseudomantis albofimbriata* mantids attacked all juvenile harlequin bugs offered over the course of the 4 days, rejected none, and consumed them in their entirety, with the occasional exception of dropped leg segments.

## Discussion

This study tested if the hibiscus harlequin bug (*Tectocoris diophthalmus*) successfully defends against predation by birds and arthropod predators. Given the chemical identity and quantity of secretions of *T. diophthalmus*, and prior literature suggesting that aldehydes should be ineffectual against birds (Staddon 1979; Aldrich 1988) but effective against arthropods (Remold 1963; Eliyahu et al. 2012), we predicted effective defense against arthropod predators but not avian predators. Surprisingly, the results of the experiments revealed the opposite pattern to our hypothesis. Our results show that harlequin bugs are defended against avian predators, but not against arthropods.

In both experiments using avian predators, harlequin bugs were protected from consumption. The effect does not appear to be due to neophobia or novelty per sé. In experiment 1, wild birds readily ate mealworms from the feeder trays, but chose not to consume or even touch the harlequin bugs, which were readily abundant in the local area and thus likely to be familiar. The subsequent use of captive birds permitted a more controlled experiment using naïve predators that have no prior exposure to harlequin bugs, and no exposure to other conspicuous or defended bugs which may induce avoidance by generalization of prior experience. While conspicuous prey can induce neophobia or avoidance in naïve birds (Lindström et al. 1999), there was no evidence of neophobia in the naïve chickens, at least for alpha males. All alphas attacked at least two bugs on first encounter, but reduced attacks over subsequent encounters (Fig. 2).

It has been demonstrated that the combination of conspicuous colors and noxious odors (Rowe and Guilford 1996) or taste (Rowe and Skelhorn 2005) can induce unlearned avoidance in domestic chicks. The pairing of bright coloration and volatile aldehyde chemicals may have enhanced the aversiveness of the harlequin bugs in this experiment. There was a partial resurgence of attacks after a 3 days hiatus in exposure to the bugs, although still fewer than first encounter (Fig. 2); this pattern would be expected for defenses based on distaste rather than illness, which should be less persistent in the memory of birds (Alcock 1970). Some evidence suggests that domestic chickens may also be repelled by aposematic coloration paired with novel (but not unpalatable) odors (Jetz et al. 2001), or even aposematic coloration alone (Schuler and Hesse 1985). It is currently unclear whether the chickens were repelled by scent, taste, coloration, or a combination of factors, but it is likely that aversion is based on unpalatability or bias rather than chemical toxicity. Future research should investigate the relative effectiveness of these aldehydes alone (in varying concentrations) compared with the visual signal alone and the combined defenses.

In contrast to the birds, mantids showed no repellency or avoidance in response to harlequin bugs. Every mantid struck at and consumed every bug during every trial. While it may be possible that four trials were not enough exposure to elicit a response, previous studies using seed bugs (Heteroptera: Lygaeidae) have successfully trained avoidance in mantids in four trials or less (Bowdish and Bultman 1993; Prudic et al. 2007). Against adult bugs, mantids shortened their latency to attack over the four trials (Fig. 3). This may be because mantids gained familiarity with this large and novel prey. If avoidance learning were occurring, a longer latency to attack would have been predicted. This experiment provided no evidence that adult harlequin bugs are defended against mantids.

Aldehydes (Remold 1963) and keto-aldehydes (Prudic et al. 2008; Eliyahu et al. 2012) should function as contact poisons for mantids. These chemicals can coat insect antennae or penetrate the cuticle directly, causing paralysis and toxicity (Remold 1963). Previous research involving keto-aldehyde defenses found that contact with these compounds can result in immediate prey rejection on first sampling (up to 80%) or death (Prudic et al. 2008). None of the four mantids tested with juvenile harlequin bugs, which produce keto-aldehydes (Staddon et al. 1987), showed evidence of rejection or illness in our study. Although the small sample size prevents a strong conclusion from being drawn from this experiment, the results suggest that juvenile harlequin bugs are not defended against mantids. It should be also noted that the giant mesquite bugs (*Thasus neocalifornicus*: Coreidae) used by Prudic et al. (2008) produce the same chemicals as harlequin bugs, but in much greater quantities. Therefore, dose is likely to be an important factor along with chemical identity in determining the success of chemical defenses against mantids and other predatory taxa.

While this experiment only systematically tested *Hierodula majuscula* and *Pseudomantis albofimbriata* mantids in the laboratory, field observations suggest additional arthropod taxa may be preying on harlequin bugs. Arthropod predators that have been documented feeding on harlequin bugs, both adults and juveniles, include assassin bugs (*Pristhesancus* sp. and *Havinthus* sp: Heteroptera: Reduviidae), lynx spiders (*Oxyopies* sp: Oxyopidae), and orb-web spiders from families Araneidae, Tetragnathidae, and Nephiladae (Fabricant, per obs). Therefore, it is likely that the laboratory findings obtained with the mantids are likely applicable to many predaceous arthropod taxa, but further experimental testing is required to confirm these observations.

There were a number of caveats to this study. In experiment 1, we cannot estimate the total number of birds that chose to ignore the bugs in the 24-h period, or confirm visitation of any bird species other than *M. melanocephala*, but this experiment does demonstrate that harlequin bugs are protected at these two sites. While it is possible that some mealworms were removed by species other than birds (e.g., possums or rats) during the overnight period, it is clear that neither mammalian nor avian predators consumed or caused damage to the harlequin bugs. In experiment 2, in contrast to alpha males, beta males showed an increase in attacks across rounds (Fig. 2). This, we suggest, is because alphas had stopped defending the bugs as a food resource, and thus, the betas were permitted to sample the bugs. Sherwin et al. (2002) found that juvenile chickens did not learn to avoid unpalatable prey by seeing the disgust reaction and subsequent rejection of prey by other chickens. Nicol (2006) suggested that in older chickens, direct experience may be more important for learning than observing others. We predict that if the experiments continued that beta males too would show a decline in interest after a similar number of experiences as the alpha males, but number of bugs available was a limiting factor in the duration of the experiment. Despite the small sample size due to bug and chicken limitations, the rapid learning of dominant alpha males is a robust result supporting avoidance learning.

## Conclusions

In our experiments, the predators of *Tectocoris diophthalmus* behaved in the exact opposite manner to our predicted outcomes, being aversive to avian predators but palatable to arthropod predators. Given that the same chemicals produced in higher concentrations can paralyze or kill arthropod predators (Remold 1963; Prudic et al. 2008), its chemical defenses likely have dose-dependent and synergistic effects (Eliyahu et al. 2012). Conversely, despite the weak defenses of the bugs, avoidance learning by birds may have been facilitated by innate biases triggered by smell or taste (Jetz et al. 2001; Rowe and Skelhorn 2005). It is important to perform behavioral assays using multiple ecologically relevant predator classes in order to declare an insect aposematic, and even this conclusion will be limited to the predatory species tested. Furthermore, the information gained in this study will make the harlequin bug a far more insightful model organism in studies of aposematism and other aspects of behavioral ecology.
